# The Comparative Effectiveness and Safety of Ambulatory Care Warfarin Management by Non-physician Providers Versus Usual Medical Care: A Systematic Review and Meta-analysis

**DOI:** 10.1177/08971900251347506

**Published:** 2025-06-02

**Authors:** Anna Sharow, Joey Champigny, John-Michael Gamble, Sherilyn K.D. Houle, Caitlin Carter, Jeff Nagge

**Affiliations:** 70382 University of Waterloo School of Pharmacy, Kitchener, ON, Canada

**Keywords:** warfarin, anti-coagulation, pharmacist

## Abstract

**Introduction:** Growing evidence suggests that non-physician providers (NPPs) can effectively and safely manage warfarin therapy. This systematic review and meta-analysis aimed to evaluate warfarin management by NPPs compared to usual medical care (UMC) in ambulatory patients. **Methods:** We conducted a systematic search of PubMed (MEDLINE), Ovid Embase, Ovid International Pharmaceutical Abstracts, Scopus, CINAHL (EBSCO), and the Cochrane Central Register of Controlled Trials (CENTRAL) from inception to January 2024. Studies were included if they were randomized controlled trials or quasi-experimental designs comparing warfarin management across professions. Two independent reviewers performed title and abstract screening, full-text review, data extraction, and risk of bias assessment. Results were pooled using random effects models. **Results:** Of 19 122 citations identified, 6 met the inclusion criteria. NPPs included pharmacists (4), nurse practitioners (1), and multidisciplinary teams (1). Meta-analysis showed no significant difference in time spent in therapeutic range (TTR) (mean difference [MD] 1.64%; 95% confidence interval [CI]-1.86 to 5.16, I^2^ = 0%)) for NPPs vs UMC. There were no differences in thrombosis (relative risk [RR] 1.23; 95% CI 0.36 to 4.23, I^2^ = 0%), hemorrhage (RR = 1.07; 95% CI 0.44 to 2.63, I^2^ = 0%), mortality (RR = 0.94; 95% CI 0.33 to 2.67, I^2^ = 0%), or patient satisfaction (standardized mean difference [SMD] 0.56; 95% CI -0.04 to 1.15, I^2^ = 85%). **Conclusion:** NPP management resulted in similar TTR as UMC. Due to few thromboembolic and hemorrhagic events, more studies are needed to determine the effects of NPP warfarin management on clinical outcomes.

## Introduction

While direct oral anticoagulants (DOACs) are now the preferred choice for most patients requiring oral anticoagulation, approximately 18% of patients still use warfarin.^1^ Due to its narrow therapeutic index, warfarin therapy requires regular monitoring of the International Normalized Ratio (INR) and dose adjustment to maintain the INR within the desired range. The need for individualization and intensive monitoring can lead to variability in the quality of care and outcomes such as thromboembolic and hemorrhagic events.^2-4^

Historically, physicians have been primarily responsible for managing warfarin therapy. However, expanding scopes of practice and the use of medical directives have enabled other health care professionals, such as nurses and pharmacists, to adopt larger roles in warfarin management. This includes clinics led by non-physician providers (NPPs), often operated by pharmacists or nurse practitioners who independently monitor and dose warfarin. Expanded scope and division of labour between different practitioners alleviates workload pressures and can help increase patient access to care.

Several studies have examined the impact of NPP management of warfarin therapy on various outcomes including time in therapeutic range (TTR), a widely accepted surrogate measure for the effectiveness and safety of warfarin management.^5-11^ Systematic reviews and meta-analyses of these studies have reported discordant results. Some suggest that NPP-managed warfarin improves TTR (ranging from 2 to 28%)^6,7,11^ compared to usual medical care (UMC), while another found no difference between modalities.^5^ The type and quality of studies included are a significant reason for the discrepancy. Hou and colleagues found that pooling results from observational studies of pharmacist-managed warfarin therapy led to 13.5% higher TTR (*P* < 0.001) compared to UMC, whereas pooling results from RCTs found no significant difference (1.3% higher, *P* = 0.548).^5^

Another limitation of existing evidence is that all previously published systematic reviews and meta-analyses in this area have focused on pharmacist management vs UMC, which ignores research involving nurse and nurse practitioner management of warfarin therapy.

Unlike some previous reviews,^5,11^ we focused our study on the management of warfarin in ambulatory settings, as dosing considerations in institutional settings differ from those in ambulatory settings due to factors such as ease of access to INR testing and patient complexity. We aimed to evaluate the comparative effectiveness (TTR, reduction in risk of thromboembolism), safety (hemorrhagic events), patient satisfaction, and cost of warfarin management by various health care professionals in ambulatory settings.

## Methods

The study was conducted in accordance with the guidelines of the Preferred Reporting Items for Systematic Reviews and Meta-Analyses (PRISMA) group. The completed PRISMA checklist is available as a supplementary appendix online. The protocol for this review has been registered with PROSPERO (CRD42018084839).^12^

### Information Sources and Search Strategy

The following databases were searched by a research librarian (CC) from inception to January 2024: PubMed(MEDLINE), Ovid Embase, Ovid International Pharmaceutical Abstracts, Scopus, CINAHL (EBSCO), and the Cochrane Central Register of Controlled Trials (CENTRAL). A grey literature search was done of the ClinicalTrials.gov database. The search strategies included a combination of both medical subject headings and keywords related to health care personnel and warfarin. Keywords were restricted to the title and abstract fields only, dependent on database functionality. All search strategies employed the use of Boolean operators, truncation and phrase searching, and results were limited to the English language. The complete search strategies for each database can be found in Online Appendix 1.

Amongst the search results, if only abstracts of research were identified, the corresponding authors were contacted with requests for full-text publications of the research, if available. Reference lists from included full-text manuscripts and the CHEST clinical practice guidelines were manually reviewed for additional citations. All database search results and additional citations were imported into Covidence (https://www.covidence.org/), where duplicates were removed.

### Eligibility Criteria

Studies were eligible for inclusion if they met each of the criteria listed in Table 1. Four reviewers were initially involved in screening (JN, SH, JMG, JD) and 2 additional reviewers (AS, JC) were added to the review in 2024. Each record was initially screened by title and abstract, followed by a full-text review. This was conducted by 2 of the 6 independent reviewers. Disagreements were resolved by consensus among reviewers. Screening was done using the Covidence platform and papers were included if they had a quasi-experimental with control or RCT design. Papers were excluded if they were conducted with a pediatric population (age <18), done in a non-ambulatory population, published in a language other than English, or if the research was published only in abstract form due to difficulty with extracting all relevant data and assessing the risk of bias. Study designs included were consistent with those recommended by the Cochrane Collaboration.^13^

**Table 1.** Eligibility Criteria.

	Inclusion Criteria	Exclusion Criteria
Population	Ambulatory care warfarin patients	Pediatric or non-ambulatory warfarin patients
Intervention	Any ambulatory warfarin care model. Models were classified based on the type of health care provider, including but not limited to: pharmacist, nurse or nurse practitioner, specialist physician, multidisciplinary team (more than 1 type of provider) without physician member, or multidisciplinary team with physician member	Practitioner not involved in dosing decisions
	Providers must have had ability to autonomously adjust warfarin dose and monitor INR	
Comparator	UMC defined as warfarin managed by a family-physician or general practitioner	No control, or a non-concurrent control during the intervention (historical control only)
Outcome	Primary outcome: TTR	No eligible outcomes
	Secondary outcomes: Major bleeding, as defined in the study; thrombosis, as defined in the study; satisfaction, assessed using an instrument with good validation properties; cost; changes in non-warfarin-related medications; all-cause emergency department visits; all-cause hospitalization; all-cause mortality; cardiovascular-related mortality	
Study design	RCT or quasi experimental with control designs	Case control design
Language	—	Non-English articles
Format	—	Abstract only

INR: international normalized ratio; TTR: time in therapeutic range; RCT: randomized controlled trial; UMC: usual medical care.

### Data Extraction

Data extraction was performed in duplicate, with each eligible record being extracted by 2 of 4 reviewers (JN, JMG, AS, JC). Each reviewer completed data collection independently from the full text of the manuscript or the supplemental materials from the studies selected for inclusion. Any inconsistencies were resolved by consensus between the 2 extracting reviewers. Extraction was done using a standardized form which was pilot-tested and modified as required to ensure all relevant data was captured. Extraction then began on a study level.

Extracted domains include study identification (author information; country; setting; funding), methods (study design), population (inclusion/exclusion criteria; patient baseline characteristics), interventions (use of decision software; method of INR determination; use of scheduling software; provision of education regarding warfarin use; assessment of adherence; systematic screening of thromboembolism or hemorrhage or warfarin adverse effects; review of medications, comorbidities, diet and drug interactions that can impact warfarin therapy), and outcomes (TTR; episodes of major hemorrhage; episodes of major thromboembolic events; cost; patient satisfaction quantifiers). During screening, all data from each outcome was sought. All results retrieved were compatible with the original outcome domain, except for TTR for 1 study, which reported this as a proportion of time spent outside of the therapeutic range.^14^ This was calculated and converted by the reviewers to TTR and reported as such.

### Risk of Bias Assessment

All included records were assessed for risk of bias by 2 of 4 reviewers (JN, JMG, AS, JC). Each reviewer performed a risk of bias assessment using the Cochrane risk of bias tool during the extraction phase. Any disagreements were resolved by consensus between the 2 reviewers involved.

### Outcomes

TTR was reported as the proportion of time in therapeutic range as defined by each RCT. Extended TTR was reported as a proportion of time in an expanded therapeutic range as defined by each RCT, typically being the prescribed INR range ±0.2. Episodes of major hemorrhage and major thromboembolic events were reported as a proportion of patients with the event. Cost was reported as the total mean cost per patient per month or as a cost-benefit ratio. Patient satisfaction quantifiers were reported as a mean score and standard deviation of the patient satisfaction quantifier used in each study.

### Data Synthesis and Analysis

A pairwise meta-analysis was conducted among included RCTs comparable in terms of their study-level characteristics including interventions, comparisons, and outcomes. Mean differences for the TTR and extended time in therapeutic range were pooled across included RCTs using a random effects model with inverse variance weighting. Pooled relative risks (RR) were calculated for studies reporting relevant dichotomous outcomes (risk of bleeding, risk of thrombosis, and risk of all-cause mortality) using random effects models. A continuity correction of 0.5 was used in studies with zero cell frequencies. A pooled standardized mean difference was calculated for patient satisfaction because of different measures used across studies for assessing overall satisfaction. Between study variance was estimated using restricted maximum likelihood estimation for all meta-analyses. We conducted a subgroup analysis whereby we stratified included RCTs based on whether study participants were stabilized on warfarin prior to randomization. We also restricted our analysis for TTR to intervention groups that included pharmacists. We planned to conduct several additional subgroup analyses to explore potential differences in results among studies with specific attributes, however, due to a limited number of studies we were unable to do so. All statistical analysis was conducted using R statistical software, version 3.6.1 (R Foundation for Statistical Computing, Vienna, Austria).

## Results

### Systematic Review

**Figure 1.**
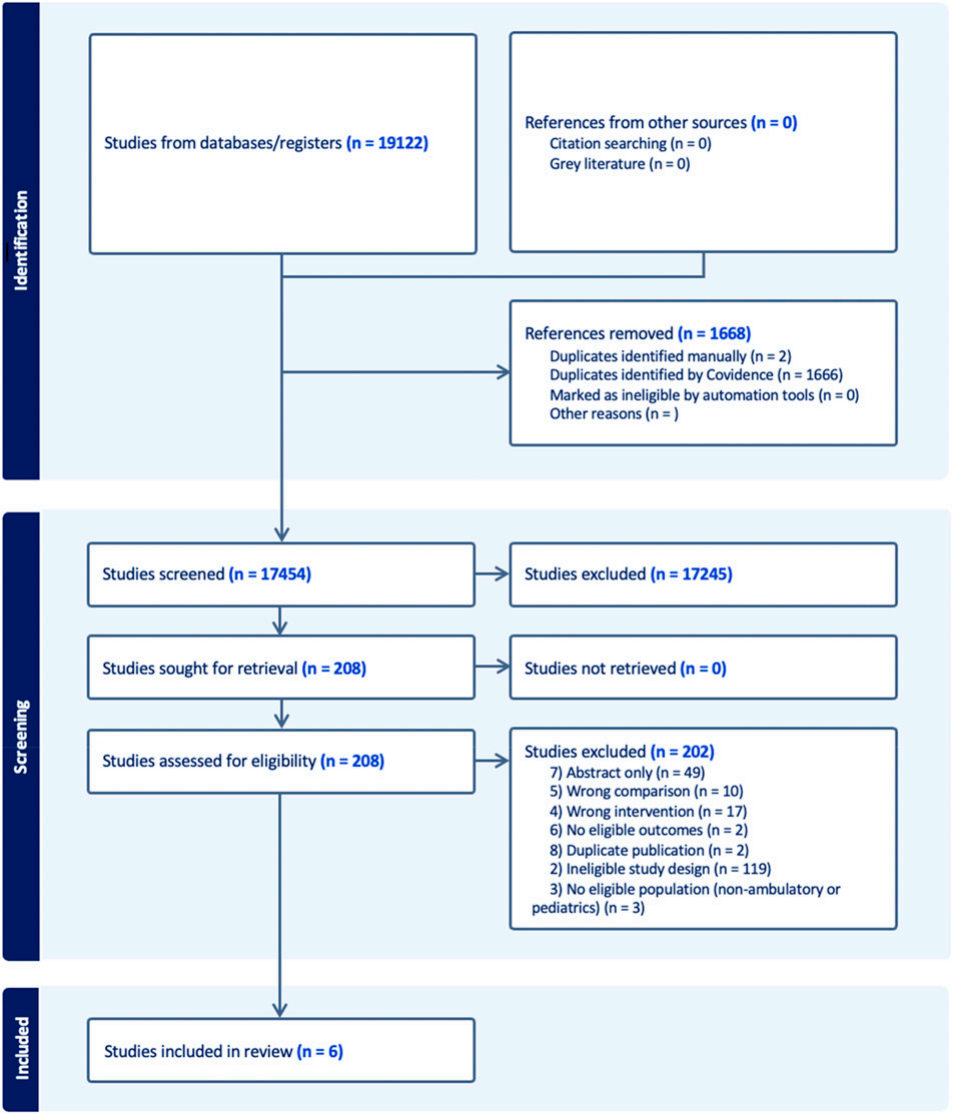
PRISMA flow diagram for included studies.

The electronic database search yielded 19 122 citations, with 17 454 remaining after removal of duplicates. Full-text review was performed on 208 publications, and 6 ultimately met the inclusion criteria. The selection process is illustrated in Figure 1, and study characteristics are detailed in Table 2. Of studies that appeared to meet eligibility criteria based on abstract and title review, 202 were excluded for the following reasons: ineligible study design (119), available as abstract only (49), incorrect intervention group (17), incorrect comparison (10), ineligible population (3), no eligible outcomes (2), or duplicates not removed by the automated system (2). There were no studies retrieved from grey literature or manual citation searching.

**Table 2.** Summary of Characteristics of Included Studies.

Study, year, country	Design	Warfarin Indications	Intervention (n)	Control (n)	Patient- years of Follow-Up	Outcomes	Results		*P* value	TTR Method
							Intervention	UMC		
Bungard, 2012, Canada^19^	RCT	Atrial fibrillation, VTE, and others. Mechanical heart valves excluded	Pharmacist management with in-person initial visit, lab INRs, and telephone follow-up (n = 32)	UMC after being educated and stabilized by pharmacist managed clinic (n = 30)	17.6 (PMA)	TTR	73.5%	76.9%	NS	RLI
					16 (UMC)					
Gray, 1985, United States^14^	Quasi-experimental (pre-post with parallel control)	MVR, AVR, atrial fibrillation, VTE and others	Pharmacist management (not defined) (n = 26)	UMC by patient’s own MD or cardiologist (n = 26)	28.7	TTR	84.5%	65.3%	<0.001	FIR
Lalonde, 2008, Canada^18^	RCT	MVR, AVR, atrial fibrillation, VTE and others	Pharmacist management via telephone (n = 128)	UMC after being educated and stabilized by pharmacist for an average of 11.3 weeks (n = 122)	69.7	TTR	77.3%	76.7%	NS	RLI
Lin, 2021, Taiwan^20^	Pre-post quasi-experimental	Atrial fibrillation, VTE, and others	Pharmacist management, POC testing, structured interview to provide education and medication review and assess adherence (n = 148)	UMC of the same patients prior to referral to the pharmacist managed clinic (n = 148)	Not reported	TTR	51.0%	78.6%	<0.001	RLI
Vadher, 1997, United Kingdom^15^	RCT	MVR, AVR, atrial fibrillation, VTE and others	Nurse practitioner management with CDSS (n = 87)	UMC by trainee MD (n = 90)	61.7	*TTR (2-3)	60.7%	50.6%	NS	RLI

AC, anticoagulation; AVR, aortic mechanical valve; CDSS, computerized decision support software; CSF, cross-section-of-the-files; FIR, fractional in range; MD, medical doctor; MMA, multidisciplinary managed anticoagulation; MVR, mechanical mitral valve; NPMA, nurse practitioner managed anticoagulation; NS, not significant; PMA, pharmacist-managed anticoagulation; POC, point-of-care; RCT, randomized controlled trial; RLI, Rosendaal linear interpolation; RNMA, nurse-managed anticoagulation; SPMA, specialist physician managed anticoagulation; UMC, usual medical care; VTE, venous thromboembolism.

*for the purposes of the meta-analysis, patients in this study were separated into Group A (therapeutic range of 2-3) and Group B (therapeutic range of 3-4.5).

#### Study characteristics

Indications for warfarin included atrial fibrillation, venous thromboembolism (VTE), mitral valve replacement (MVR), aortic valve replacement (AVR), and others. Mean patient-years of follow was 41.5. Four studies were RCTs, and 2 were quasi-experimental with control designs. Four studies compared usual medical care (UMC) to pharmacist-managed anticoagulation (PMA),^14,17-19^ 1 compared UMC to a multidisciplinary managed anticoagulation clinic (MMC) with a hematologist and pharmacist,^16^ and 1 compared medical trainee provided anticoagulation to nurse practitioner managed anticoagulation (NPMA).^15^ We agreed by consensus to consider medical trainees as UMC because they were under the supervision of general physicians. Three intervention groups reported using computerized dosing support^15^ or dosage adjustment protocols,^16^ while others did not mention the use of dosing tools. Two studies^19^ utilized point-of-care INR testing in the intervention group. All patients in 3 studies received standardized education and had their warfarin dose stabilized before randomization.^16-18^ In 1 study, patients received standardized education but did not have dose stabilization before group assignment.^19^

#### TTR Results

Of the 6 studies reporting on TTR, 2 found statistically significant benefits favouring NPP care.^14,19^

#### Thromboembolism and Hemorrhage Results

Five studies reported on thrombotic and hemorrhagic events, but these outcomes were rare.^15-18^ In 1 study, 1 hemorrhagic event was observed within the NPP group (n = 26) and 3 were observed within the UMC group (n = 26), along with no reported thrombotic events in the NPP group compared to 4 in the UMC group.^14^ These events were seen at similar rates across all the studies which reported on these outcomes. Three studies reported on death and found that events occurred at similar rates between groups.^16,17^

#### Patient Satisfaction and Cost Results

All studies that reported on patient satisfaction found benefits associated with being in the intervention (ie, NPP) group.^16-19^ One study reported on the costs of therapy and found that pharmacist-managed care was associated with a decrease in cost.^14^ In 1 study, a cost-benefit analysis showed a benefit-to-cost ratio of 6.55, indicating that the benefits of the NPP service outweigh the costs.^14^ Insufficient details were provided to confirm this finding.

#### Risk of Bias in Included Studies

The risk of bias in the included studies was varied and is summarized in Table 3. Briefly, most studies had a low risk of bias for sequence generation and a low or unclear risk for allocation concealment. Blinding of outcome assessors was generally low or unclear. Selective outcome reporting could not be assessed for almost all included studies since protocols were not available. Notably, the risk of bias from blinding of participants and personnel was high in almost all included studies. Several studies had additional sources of bias:

(1) Gray et al.^14^: The risk of bias was increased due to the use of medical residents in the control group, potentially leading to suboptimal warfarin management given their relative clinical inexperience.
(2) Vadher et al.^15^: The risk of bias was high due to the inclusion of physician members from the hospital anticoagulation clinic in the control group, which would not resemble typical UMC.
(3) Lin et al.^19^: The risk of bias was high due to the inclusion criteria allowing only previously unstable or unaware patients to be included in the NPP group.

**Table 3.** Risk of Bias in Included Studies.

Study	Sequence Generation	Allocation Concealment	Blinding of Participants & Personnel	Blinding of Outcome Assessors	Incomplete Outcome Data	Selective Outcome Reporting	Other
LaLonde 2008^18^	Low	Low	High	Low	Low	Unclear	Low
Wilson 2003^16^	Low	Low	High	Low	Low	Unclear	Unclear
Vadher 1997^15^	Low	Unclear	High	Unclear	Unclear	Unclear	High
Gray 1985^14^	High	High	High	High	Low	Unclear	High
Bungard 2012^19^	Low	Low	High	Unclear	Low	Low	Low
Lin 2021^20^	High	High	High	High	Higher	Unclear	High

### Meta-analysis

**Figure 2.**
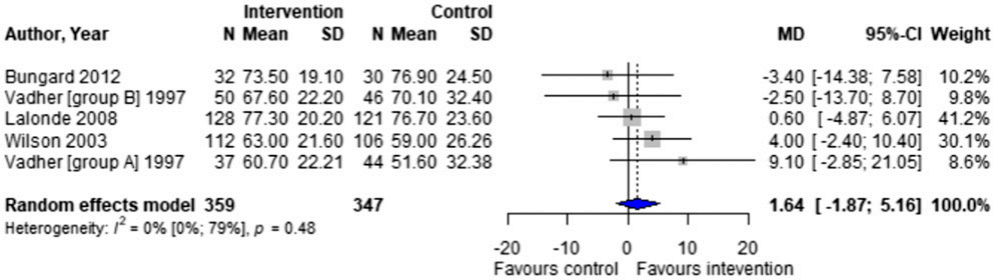
Forest plots for effectiveness and safety outcomes: TTR comparing UMC (control) and NPP (intervention). NPPs: Non-physician providers; TTR: time in therapeutic range; UMC: usual medical care.

**Figure 3.**
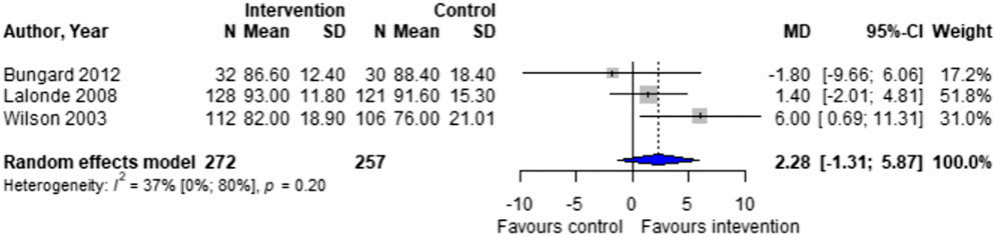
Forest plots for effectiveness and safety outcomes: eTTR comparing UMC (control) and NPP (intervention). NPPs: Non-physician providers; UMC: usual medical care.

**Figure 4.**
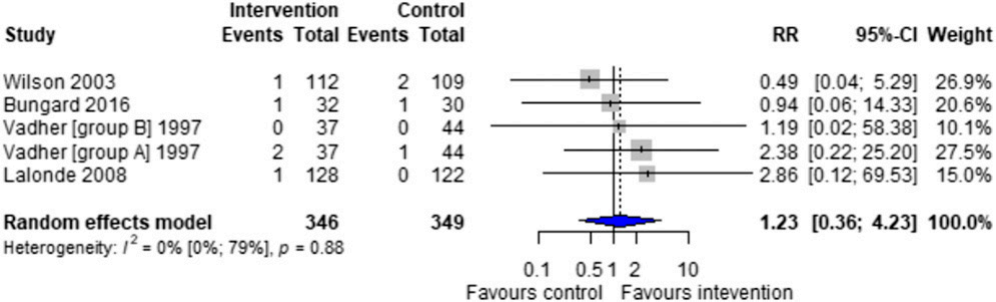
Forest plots for effectiveness and safety outcomes: thrombotic events comparing UMC (control) and NPP (intervention). NPPs: Non-physician providers; UMC: usual medical care.

**Figure 5.**
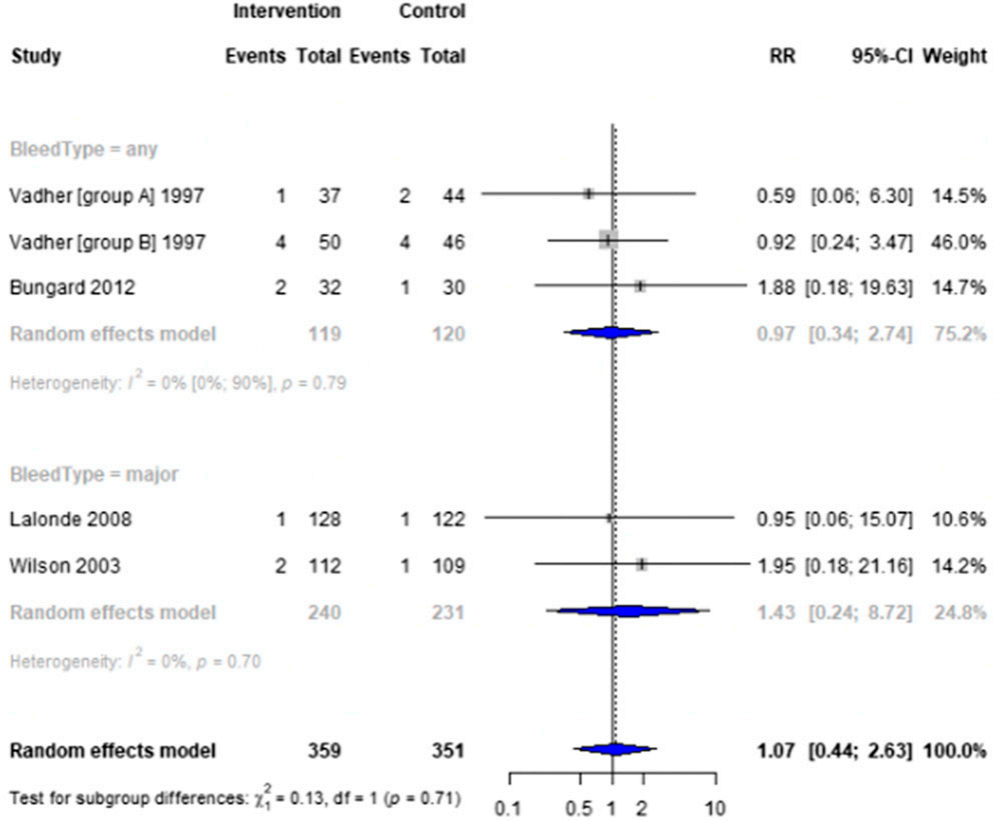
Forest plots for effectiveness and safety outcomes: hemorrhagic events comparing UMC (control) and NPP (intervention). NPPs: Non-physician providers; UMC: usual medical care.

**Figure 6.**
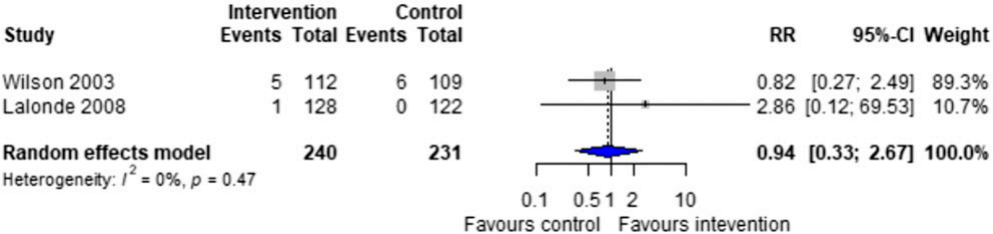
Forest plots for effectiveness and safety outcomes: death comparing UMC (control) to NPP (intervention). NPPs: Non-physician providers; UMC: usual medical care.

**Figure 7.**
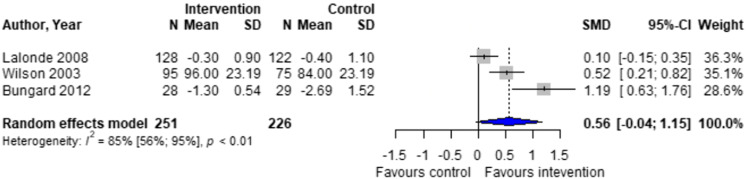
Forest plots for effectiveness and safety outcomes: for patient satisfaction comparing UMC (control) and NPP (intervention). NPPs: Non-physician providers; UMC: usual medical care.

**Figure 8.**
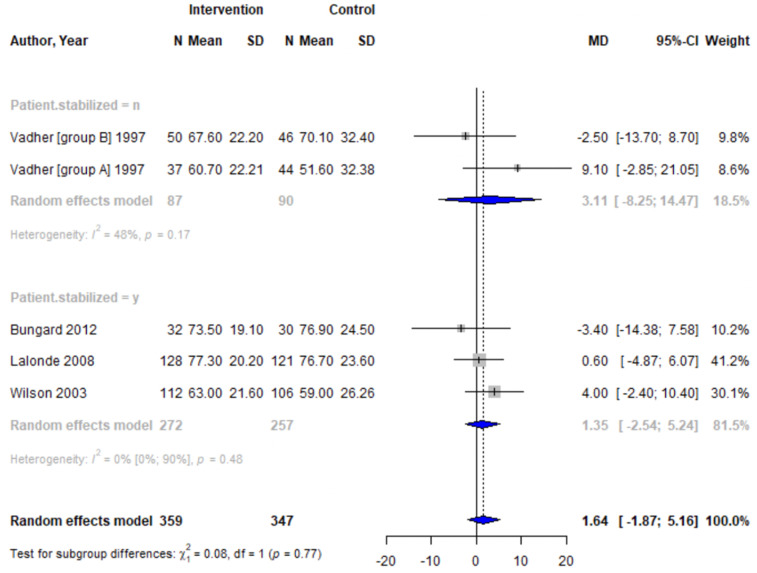
Forest plot for subgroup analysis on the effect of patient stabilization prior to analysis comparing UMC (control) and NPP (intervention) for patients who were not stabilized (top plot) and patients who were stabilized (middle plot) prior to study enrolment. NPPs: Non-physician providers; UMC: usual medical care.

Forest plots for effectiveness and safety outcomes are shown in Figures 2–7. Of the 6 included studies, 2 were excluded from the meta-analysis due to methodological differences (2 were not RCTs).^14,19^ Due to the limited number of studies included we were unable to perform a network meta-analysis. Additionally, there were too few patients and insufficient details provided regarding the specifics of the NPP services to perform many of the preplanned subgroup analyses. The exception to this was the subgroup analysis which looked for differences seen in TTR between patients who were and were not stabilized prior to analysis in studies. The forest plot for this analysis is shown in Figure 8.

#### Effect of Intervention on TTR

Four studies reported TTR. There was no significant difference in TTR (mean difference [MD] 1.64%; 95% confidence interval [CI]-1.86 to 5.16, I^2^ = 0%) between NPP and UMC. Studies including pharmacists in the intervention arm showed consistent results (MD 1.35; 95% CI -2.54 to 5.24, I^2^ = 0%). Similarly, no significant difference was observed in the 4 studies reporting on time in the expanded therapeutic range (1.7 to 3.3) (MD = 2.28%; 95% CI – 1.31 to 5.87, I^2^ = 36.9%).^16-18^

#### Effect of Intervention on Risk of Thrombosis

Risk of thromboembolic events, recorded if the event resulted in hospitalization or emergency department visit, showed no significant difference between NPP and UMC in the 4 studies reporting this outcome (relative risk [RR] 1.23; 95% CI 0.36 to 4.23, I^2^ = 0%). The number of events across the included studies was low, with each study having between zero and 2 events.^15-18^

#### Effect of Intervention on Risk of Hemorrhage and Death

Four studies reported on the risk of hemorrhage, generally defined as hemorrhage resulting in hospitalization or emergency department visit,^15,18^ though 1 study did specify this definition.^16^ Another study classified hemorrhages by their location and severity (fatal, life-threatening, or potentially life-threatening).^17.^There was no significant difference in the risk of hemorrhage between NPP and UMC when combining results from the 5 studies reporting this outcome (RR = 1.07; 95% CI 0.44 to 2.63, I^2^ = 0%).^15-18^ Two studies reported on the risk of death, finding no difference between NPP and UMC (RR = 0.94; 95% CI 0.33 to 2.67, I^2^ = 0%).^16,17^ The number of events reported was low, with the highest number of events being 4 in 1 study.^15^

#### Effect of Intervention on Patient Satisfaction

There was a non-significant higher patient satisfaction in the NPP group compared to UMC in the 3 studies reporting this outcome (standardized mean difference [SMD] 0.56; 95% CI -0.04 to 1.15, I^2^ = 85%).^16-18^ Notably, there is significant heterogeneity in this outcome, possibly due to differences in measurement scales used across studies.

#### Subgroup Analysis

Subgroup analysis was performed to detect any differences in TTR between patients who were stabilized prior to enrolment in the study. The meta-analysis showed no significant difference in TTR between groups who were stabilized prior to undergoing assignment (3 studies, MD = 1.35; 95% CI -2.54 to 5.24) and those who were not (2 studies, MD = 3.1; 95% CI -8.26 to 14.47).

## Discussion

Our study has 3 primary findings. First, there are few RCTs comparing the effectiveness and safety of different anticoagulation management models. Second, pooled results from these studies were similar between NPP and UMC for the surrogate measure, TTR, and patient satisfaction. Furthermore, subgroup analysis of prior stabilization effects revealed no significant differences, indicating that NPPs can effectively manage all warfarin patients, regardless of their stability. Third, due to the limited number of events, there is insufficient evidence to determine differences in clinical outcomes, such as hemorrhagic, thromboembolic, and mortality rates between groups.

Comparing our findings with several other meta-analyses and systematic reviews reveals some differences. Saokaew et al^20^ performed a meta-analysis of 24 studies comparing pharmacist-participated warfarin therapy (PPWT) with usual care. They found a significant reduction in total hemorrhage [RR, 0.51; 95% CI, 0.28 to 0.94] but no effect on thromboembolic events, major hemorrhage, or mortality associated with PPWT compared to usual care. These results may differ from ours because the Saokaew review included studies conducted in non-ambulatory settings, and also included several observational studies, impacting the overall quality of evidence as observational studies generally have lower quality and are at greater risk of bias. The inclusion of non-ambulatory settings limits the direct applicability of these findings to the primary care setting and may also explain the significant reduction in total hemorrhage, since close monitoring of INRs during periods of instability, such as acute illness requiring inpatient care, likely contributed to the improved ability to prevent such events.

Mansoor et al performed a systematic review of the quality of anticoagulation services provided by pharmacists in outpatient settings.^21^ Their findings included higher TTR (22 of 25 studies), lower or equal risk of hemorrhage (10 of 12 studies) and thromboembolic events (9 of 10 studies), lower rates of hospitalization or emergency department visits (9 of 9 studies), and lower cost of therapy (6 of 6 studies) in pharmacist managed anticoagulation groups compared to routine medical care.^21^ Similar to Saokaew et al, these findings likely differ from ours due to the inclusion of mostly observational studies, with only three of 25 studies being RCTs.^21^ The only other meta-analysis focusing solely on RCTs of pharmacist-managed warfarin therapy found significant benefits for patient satisfaction but no significant improvements in expanded TTR, thrombotic events, hemorrhagic events, or mortality, consistent with our findings.^11^ Differences in study design, such as inclusion criteria not requiring autonomous dosing by the non-UMC group, focusing on pharmacists only, and the inclusion of non-ambulatory patients, limit direct comparisons to our study.

Our meta-analysis has several limitations, both inherent to the individual studies included and arising from the review process itself. Many studies had small numbers of patients, limiting our ability to evaluate outcomes associated with infrequent events, such as thromboembolism and hemorrhage. Additionally, only 1 included study reported on cost^14^ and the findings should be interpreted cautiously due to insufficient details provided. There was also limited information regarding the delivery of care for patients in the NPP groups. Details concerning specific interventions were sparse and varied between studies, including differences in provider training, patient education, dose adjustment protocols, and INR measurement methods. For example, some studies followed approved protocols for warfarin dose adjustments,^15,19^ which are associated with higher TTR.^22^ Furthermore, variability in the methods used for INR determination with 2 studies using point-of-care (POC) devices, introduces complexity in interpreting whether observed elevations in TTR were attributable to enhanced management by the practitioner or the utilization of POC devices, which may improve TTR by approximately 6%.^19,23^ There were also inconsistencies in TTR calculation methods. Most included studies used the Rosendaal method, which provides a continuous measurement of INR values, whereas 1 study used the fractional in-range method, potentially leading to a higher reported TTR.^14,24^ Additionally, varying definitions of endpoints, such as thrombosis and hemorrhage, made direct comparisons and drawing conclusions difficult. Finally, only events which resulted in ED visits or hospitalization were included, which may have led to under-reporting outcomes which were managed in an ambulatory setting.

Bias was an issue for the included studies, with several demonstrating a high risk of bias across multiple criteria. The lack of blinding in all studies may have influenced patient satisfaction reporting. The tools used to assess patient satisfaction varied across studies, with 1 study using a non-validated tool, potentially affecting the reliability of these results.^19^ These limitations may help explain some of the heterogeneity (I^2^ = 85%) seen across studies reporting on patient satisfaction. While the magnitude of these results should be interpreted cautiously, the direction of effect on patient satisfaction consistently favoured NPP management. Furthermore, some studies allowed or required physician consultation in certain cases, which may have impacted the measured effects attributed to NPP management. For example, 1 study allowed the NPP group to seek a physician’s opinion if they were unsure how to manage an INR,^15^ while another required physician intervention in the case of elevated INR.^19^ Finally, grouping all providers into a single UMC or NPP category posed additional challenges. However, grouping all non-physician providers into 1 category was ultimately determined to be appropriate given that the outcomes were found to be similar among each of the providers in the studies.

When considering the limitations of our results at the level of the review process, several factors stemmed from the limited number of patients. First, we were unable to perform network meta-analysis, which restricts the ability to make comparisons across the different intervention groups. The limited number of patients also meant we had inadequate additional information for most of the pre-planned subgroup analyses. Consequently, the findings from the subgroup analysis that we did perform should be viewed with caution, as this was not prespecified. Furthermore, only 1 RCT reported on cost, which prevented a meta-analysis from being performed for this outcome.^14^ Finally, restricting our review to studies published in English only may have led to the exclusion of articles that would have otherwise met all other inclusion and exclusion criteria.

Future research into the safety and effectiveness of NPP management of warfarin therapy in ambulatory adult patients should use the Rosendaal method when calculating TTR. While the method has certain limitations, such as its reliance on interpolation between measurements, it remains the most widely accepted and commonly used approach.^25^ Other interventions, like POC testing devices and decision support tools, should also be used to ensure that warfarin therapy is being managed optimally. Similar analyses should be conducted periodically to include new data from studies with larger sample sizes, which may improve the detection of rare outcomes such as hemorrhage and thrombosis.

## Conclusion

Non-physician providers (NPPs) achieved similar time in therapeutic range (TTR) compared to usual medical care (UMC), indicating comparable effectiveness in this surrogate measure of the quality of warfarin management. Due to the low rates of thromboembolism and hemorrhage observed, definitive conclusions about comparative clinical effectiveness and safety cannot be drawn at this time. Patient satisfaction favoured the NPP group compared to UMC, though findings were not significant. These findings support the role of NPPs in warfarin management by demonstrating their potential to enhance patient experience and maintain effectiveness.

## Supplemental Material


Supplemental Material – The Comparative Effectiveness and Safety of Ambulatory Care Warfarin Management by Non-physician Providers Versus Usual Medical Care: A Systematic Review and Meta-analysis
Supplemental Material for The Comparative Effectiveness and Safety of Ambulatory Care Warfarin Management by Non-physician Providers Versus Usual Medical Care: A Systematic Review and Meta-analysis by Anna Sharow, Joey Champigny, JM Gamble, Sherilyn Houle, Caitlin Carter, and Jeff Nagge in Journal of Pharmacy Practices

## Data Availability

All data analyzed in this study were obtained from publicly available published sources, as cited in the manuscript. Extracted data used for analysis are available from the corresponding author upon reasonable request.*
