# Supplementary material for: The Comparative Effectiveness and Safety of Ambulatory Care Warfarin Management by Non-physician Providers Versus Usual Medical Care: A Systematic Review and Meta-analysis
Source: J Pharm Pract. 2025 Jun 2;38(6):540–50. doi: 10.1177/08971900251347506 (PMC12518879; doi:10.1177/08971900251347506)
Supplement: Supplemental Material – The Comparative Effectiveness and Safety of Ambulatory Care Warfarin Management by Non-physician Providers Versus Usual Medical Care: A Systematic Review and Meta-analysis [file sj-pdf-1-jpp-10.1177_08971900251347506.pdf]

Search strategies:

PubMed(MEDLINE)

(pharmacists[mesh] OR pharmacist*[tiab] OR pharmacy[mesh] OR pharmacy[tiab] OR pharmacies[mesh] OR pharmacies[tiab] OR community pharmacy services[mesh] OR nurses[mesh] OR nurse*[tiab] OR nurse practitioners[mesh] OR nurse practitioner*[tiab] OR physicians[mesh] OR physician*[tiab] OR "usual care"[tiab] OR "routine care"[tiab] OR "standard care"[tiab] OR multidisciplinary[tiab] OR multi-disciplinary[tiab] OR interdisciplinary[tiab] OR inter-disciplinary[tiab] OR "anticoagulation management service"[tiab] OR allied health personnel[mesh] OR "allied health"[tiab]) **AND** (warfarin[mesh] OR warfarin[tiab] OR coumadin[tiab] OR coumadine[tiab] OR adoisine[tiab] OR aldocumar[tiab] OR befarin[tiab] OR carfin[tiab] OR circuvit[tiab] OR coumadan[tiab] OR coumafene[tiab] OR coumaphene[tiab] OR dagonal[tiab] OR farin[tiab] OR jantoven[tiab] OR kumatox[tiab] OR maforan[tiab] OR marevan[tiab] OR orfarin[tiab] OR prothromadin[tiab] OR sofarin[tiab] OR tedicumar[tiab] OR tintorane[tiab] OR uniwarfin[tiab] OR warfant[tiab] OR waran[tiab] OR warfar[tiab] OR warfarine[tiab] OR warfil[tiab] OR warfilone[tiab] OR warnerin[tiab] OR "antivitamin K"[tiab] OR "antivitamins K"[tiab] OR vitamin K antagonist*[tiab]) **AND** English[lang]

Ovid Embase

1 exp pharmacist/
2 pharmacist*.ti,ab.
3 exp pharmacy/
4 pharmacy.ti,ab.
5 pharmacies.ti,ab.
6 exp nurse/
7 nurse*.ti,ab.
8 exp nurse practitioner/
9 nurse practitioner*.ti,ab.
10 exp physician/
11 physician*.ti,ab.
12 usual care.ti,ab.
13 routine care.ti,ab.
14 standard care.ti,ab.
15 multidisciplinary.ti,ab.
16 multi-disciplinary.ti,ab.
17 interdisciplinary.ti,ab.
18 inter-disciplinary.ti,ab.
19 anticoagulation management service.ti,ab.
20 exp paramedical personnel/
21 allied health.ti,ab.
22 or/1-21
23 exp warfarin/
24 warfarin.ti,ab.
25 coumadin.ti,ab.
26 coumadine.ti,ab.
27 adoisine.ti,ab.
28 aldocumar.ti,ab.

29 befarin.ti,ab.
30 carfin.ti,ab.
31 circuvit.ti,ab.
32 coumadan.ti,ab.
33 coumafene.ti,ab.
34 coumaphene.ti,ab.
35 dagonal.ti,ab.
36 farin.ti,ab.
37 jantoven.ti,ab.
38 kumatox.ti,ab.
39 maforan.ti,ab.
40 marevan.ti,ab.
41 orfarin.ti,ab.
42 prothromadin.ti,ab.
43 sofarin.ti,ab.
44 tintorane.ti,ab.
45 tedicumar.ti,ab.
46 uniwarfin.ti,ab.
47 warfant.ti,ab.
48 waran.ti,ab.
49 warfar.ti,ab.
50 warfarine.ti,ab.
51 warfil.ti,ab.
52 warfilone.ti,ab.
53 warnerin.ti,ab.
54 exp antivitamin K/
55 antivitamin* K.ti,ab.
56 vitamin K antagonist*.ti,ab.
57 or/23-56
58 22 and 57
59 limit 58 to english language

****

Ovid IPA

1 pharmacist*.ti,ab.
2 pharmacy.ti,ab.
3 pharmacies.ti,ab.
4 nurse*.ti,ab.
5 nurse practitioner*.ti,ab.
6 physician*.ti,ab.
7 usual care.ti,ab.
8 routine care.ti,ab.
9 standard care.ti,ab.
10 multidisciplinary.ti,ab.
11 multi-disciplinary.ti,ab.

12 interdisciplinary.ti,ab.
13 inter-disciplinary.ti,ab.
14 anticoagulation management service.ti,ab.
15 allied health.ti,ab.
16 or/1-15
17 warfarin.ti,ab.
18 coumadin.ti,ab.
19 coumadine.ti,ab.
20 adoisine.ti,ab.
21 aldocumar.ti,ab.
22 befarin.ti,ab.
23 carfin.ti,ab.
24 circuvit.ti,ab.
25 coumadan.ti,ab.
26 coumafene.ti,ab.
27 coumaphene.ti,ab.
28 dagonal.ti,ab.
29 farin.ti,ab.
30 jantoven.ti,ab.
31 kumatox.ti,ab.
32 maforan.ti,ab.
33 marevan.ti,ab.
34 orfarin.ti,ab.
35 prothromadin.ti,ab.
36 sofarin.ti,ab.
37 tintorane.ti,ab.
38 tedicumar.ti,ab.
39 uniwarfin.ti,ab.
40 warfant.ti,ab.
41 waran.ti,ab.
42 warfar.ti,ab.
43 warfarine.ti,ab.
44 warfil.ti,ab. (0)
45 warfilone.ti,ab.
46 warnerin.ti,ab.
47 antivitamin* K.ti,ab.
48 vitamin K antagonist*.ti,ab.
49 or/17-48
50 16 and 49
51 limit 50 to english language

****

Scopus

((TITLE-ABS (pharmacist* OR pharmacy OR pharmacies) OR TITLE-ABS (nurse* OR "nurse practitioner" OR "nurse practitioners" OR physician*) OR TITLE-ABS ("usual care" OR "routine care"

OR "standard care") OR TITLE-ABS (multidisciplinary OR multi-disciplinary OR interdisciplinary OR inter-disciplinary) OR TITLE-ABS ("anticoagulation management service" OR "allied health"))) **AND** ((TITLE-ABS (warfarin OR coumadin OR coumadine OR adoisine OR aldocumar OR befarin OR carfin OR circuvit OR coumadan OR coumafene OR coumaphene OR dagonal OR farin OR jantoven OR kumatox OR maforan OR marevan OR orfarin OR prothromadin) OR TITLE-ABS (sofarin OR tedicumar OR tintorane OR uniwarfin OR waran OR warfant OR warfar OR warfarine OR warfil OR warfilone OR warnerin OR "antivitamin K" OR "antivitamins K" OR "vitamin K antagonist" OR "vitamin K antagonists"))) **AND** (LIMIT-TO (LANGUAGE , "English "))

Cochrane CENTRAL

#1 ([mh pharmacists] or pharmacist* or [mh pharmacy] or pharmacy or [mh pharmacies] or pharmacies or [mh "community pharmacy services"] or [mh nurses] or nurse* or [mh "nurse practitioners"] or nurse practitioner* or [mh physicians] or physician* or "usual care" or "routine care" or "standard care" or multidisciplinary or multi-disciplinary or interdisciplinary or inter-disciplinary or "anticoagulation management service" or [mh "allied health personnel"] or "allied health")

#2 ([mh warfarin] or warfarin or coumadin or coumadine or adoisine or aldocumar or befarin or carfin or circuvit or coumadan or coumafene or coumaphene or dagonal or farin or jantoven or kumatox or maforan or marevan or orfarin or prothromadin or sofarin or tedicumar or tintorane or uniwarfin or warfant or waran or warfar or warfarine or warfil or warfilone or warnerin or "antivitamin K" or "antivitamins K" or "vitamin K antagonist" or "vitamin K antagonists")

#3 #1 and #2

CINAHL (EBSCO)

#	Query	Limiters/Expanders	Last Run Via
S62	S24 AND S60	Limiters - English Language<br>Search modes - Boolean/Phrase	Interface - EBSCOhost Research Databases<br>Search Screen - Advanced Search<br>Database - CINAHL
S61	S24 AND S60	Search modes - Boolean/Phrase	Interface - EBSCOhost Research Databases

			Search Screen - Advanced Search<br>Database - CINAHL
S60	S25 OR S26 OR S27 OR S28 OR S29 OR S30 OR S31 OR S32 OR S33 OR S34 OR S35 OR S36 OR S37 OR S38 OR S39 OR S40 OR S41 OR S42 OR S43 OR S44 OR S45 OR S46 OR S47 OR S48 OR S49 OR S50 OR S51 OR S52 OR S53 OR S54 OR S55 OR S56 OR S57 OR S58 OR S59	Search modes - Boolean/Phrase	Interface - EBSCOhost Research Databases<br>Search Screen - Advanced Search<br>Database - CINAHL
S59	TX "vitamin K antagonists"	Search modes - Boolean/Phrase	Interface - EBSCOhost Research Databases<br>Search Screen - Advanced Search<br>Database - CINAHL
S58	TX "vitamin K antagonist"	Search modes - Boolean/Phrase	Interface - EBSCOhost Research Databases<br>Search Screen - Advanced Search<br>Database - CINAHL
S57	TX "antivitamins K"	Search modes - Boolean/Phrase	Interface - EBSCOhost Research Databases<br>Search Screen - Advanced Search<br>Database - CINAHL
S56	TX "antivitamin K"	Search modes - Boolean/Phrase	Interface - EBSCOhost Research

			Databases Search Screen - Advanced Search Database - CINAHL
S55	TX warnerin	Search modes - Boolean/Phrase	Interface - EBSCOhost Research Databases Search Screen - Advanced Search Database - CINAHL
S54	TX warfilone	Search modes - Boolean/Phrase	Interface - EBSCOhost Research Databases Search Screen - Advanced Search Database - CINAHL
S53	TX warfil	Search modes - Boolean/Phrase	Interface - EBSCOhost Research Databases Search Screen - Advanced Search Database - CINAHL
S52	TX warfarine	Search modes - Boolean/Phrase	Interface - EBSCOhost Research Databases Search Screen - Advanced Search Database - CINAHL
S51	TX warfar	Search modes - Boolean/Phrase	Interface - EBSCOhost

			Research Databases Search Screen - Advanced Search Database - CINAHL
S50	TX waran	Search modes - Boolean/Phrase	Interface - EBSCOhost Research Databases Search Screen - Advanced Search Database - CINAHL
S49	TX warfant	Search modes - Boolean/Phrase	Interface - EBSCOhost Research Databases Search Screen - Advanced Search Database - CINAHL
S48	TX uniwarfin	Search modes - Boolean/Phrase	Interface - EBSCOhost Research Databases Search Screen - Advanced Search Database - CINAHL
S47	TX tedicumar	Search modes - Boolean/Phrase	Interface - EBSCOhost Research Databases Search Screen - Advanced Search Database - CINAHL
S46	TX tintorane	Search modes -	Interface -

		Boolean/Phrase	EBSCOhost Research Databases Search Screen - Advanced Search Database - CINAHL
S45	TX sofarin	Search modes - Boolean/Phrase	Interface - EBSCOhost Research Databases Search Screen - Advanced Search Database - CINAHL
S44	TX prothromadin	Search modes - Boolean/Phrase	Interface - EBSCOhost Research Databases Search Screen - Advanced Search Database - CINAHL
S43	TX orfarin	Search modes - Boolean/Phrase	Interface - EBSCOhost Research Databases Search Screen - Advanced Search Database - CINAHL
S42	TX marevan	Search modes - Boolean/Phrase	Interface - EBSCOhost Research Databases Search Screen - Advanced Search Database - CINAHL

S41	TX maforan	Search modes - Boolean/Phrase	Interface - EBSCOhost Research Databases Search Screen - Advanced Search Database - CINAHL
S40	TX kumatox	Search modes - Boolean/Phrase	Interface - EBSCOhost Research Databases Search Screen - Advanced Search Database - CINAHL
S39	TX jantoven	Search modes - Boolean/Phrase	Interface - EBSCOhost Research Databases Search Screen - Advanced Search Database - CINAHL
S38	TX farin	Search modes - Boolean/Phrase	Interface - EBSCOhost Research Databases Search Screen - Advanced Search Database - CINAHL
S37	TX dagonal	Search modes - Boolean/Phrase	Interface - EBSCOhost Research Databases Search Screen - Advanced Search Database -

			CINAHL
S36	TX coumaphene	Search modes - Boolean/Phrase	Interface - EBSCOhost Research Databases Search Screen - Advanced Search Database - CINAHL
S35	TX coumafene	Search modes - Boolean/Phrase	Interface - EBSCOhost Research Databases Search Screen - Advanced Search Database - CINAHL
S34	TX coumadan	Search modes - Boolean/Phrase	Interface - EBSCOhost Research Databases Search Screen - Advanced Search Database - CINAHL
S33	TX circuvit	Search modes - Boolean/Phrase	Interface - EBSCOhost Research Databases Search Screen - Advanced Search Database - CINAHL
S32	TX carfin	Search modes - Boolean/Phrase	Interface - EBSCOhost Research Databases Search Screen - Advanced Search

			Database - CINAHL
S31	TX befarin	Search modes - Boolean/Phrase	Interface - EBSCOhost Research Databases Search Screen - Advanced Search Database - CINAHL
S30	TX aldocumar	Search modes - Boolean/Phrase	Interface - EBSCOhost Research Databases Search Screen - Advanced Search Database - CINAHL
S29	TX adoisine	Search modes - Boolean/Phrase	Interface - EBSCOhost Research Databases Search Screen - Advanced Search Database - CINAHL
S28	TX coumadine	Search modes - Boolean/Phrase	Interface - EBSCOhost Research Databases Search Screen - Advanced Search Database - CINAHL
S27	TX coumadin	Search modes - Boolean/Phrase	Interface - EBSCOhost Research Databases Search Screen - Advanced

			Search Database - CINAHL
S26	TX warfarin	Search modes - Boolean/Phrase	Interface - EBSCOhost Research Databases Search Screen - Advanced Search Database - CINAHL
S25	(MH "Warfarin")	Search modes - Boolean/Phrase	Interface - EBSCOhost Research Databases Search Screen - Advanced Search Database - CINAHL
S24	S1 OR S2 OR S3 OR S4 OR S5 OR S6 OR S7 OR S8 OR S9 OR S10 OR S11 OR S12 OR S13 OR S14 OR S15 OR S16 OR S17 OR S18 OR S19 OR S20 OR S21 OR S22 OR S23	Search modes - Boolean/Phrase	Interface - EBSCOhost Research Databases Search Screen - Advanced Search Database - CINAHL
S23	TX "allied health"	Search modes - Boolean/Phrase	Interface - EBSCOhost Research Databases Search Screen - Advanced Search Database - CINAHL
S22	(MH "Allied Health Personnel+")	Search modes - Boolean/Phrase	Interface - EBSCOhost Research Databases Search Screen -

			Advanced Search Database - CINAHL
S21	TX "anticoagulation management service"	Search modes - Boolean/Phrase	Interface - EBSCOhost Research Databases Search Screen - Advanced Search Database - CINAHL
S20	TX inter-disciplinary	Search modes - Boolean/Phrase	Interface - EBSCOhost Research Databases Search Screen - Advanced Search Database - CINAHL
S19	TX interdisciplinary	Search modes - Boolean/Phrase	Interface - EBSCOhost Research Databases Search Screen - Advanced Search Database - CINAHL
S18	TX multi-disciplinary	Search modes - Boolean/Phrase	Interface - EBSCOhost Research Databases Search Screen - Advanced Search Database - CINAHL
S17	TX multidisciplinary	Search modes - Boolean/Phrase	Interface - EBSCOhost Research Databases

			Search Screen - Advanced Search Database - CINAHL
S16	(MH "Multidisciplinary Care Team+")	Search modes - Boolean/Phrase	Interface - EBSCOhost Research Databases Search Screen - Advanced Search Database - CINAHL
S15	TX "standard care"	Search modes - Boolean/Phrase	Interface - EBSCOhost Research Databases Search Screen - Advanced Search Database - CINAHL
S14	TX "routine care"	Search modes - Boolean/Phrase	Interface - EBSCOhost Research Databases Search Screen - Advanced Search Database - CINAHL
S13	TX "usual care"	Search modes - Boolean/Phrase	Interface - EBSCOhost Research Databases Search Screen - Advanced Search Database - CINAHL
S12	TX physician*	Search modes - Boolean/Phrase	Interface - EBSCOhost Research

			Databases Search Screen - Advanced Search Database - CINAHL
S11	(MH "Physicians+")	Search modes - Boolean/Phrase	Interface - EBSCOhost Research Databases Search Screen - Advanced Search Database - CINAHL
S10	TX nurse practitioner*	Search modes - Boolean/Phrase	Interface - EBSCOhost Research Databases Search Screen - Advanced Search Database - CINAHL
S9	(MH "Nurse Practitioners+")	Search modes - Boolean/Phrase	Interface - EBSCOhost Research Databases Search Screen - Advanced Search Database - CINAHL
S8	TX nurse*	Search modes - Boolean/Phrase	Interface - EBSCOhost Research Databases Search Screen - Advanced Search Database - CINAHL
S7	(MH "Nurses+")	Search modes - Boolean/Phrase	Interface - EBSCOhost

			Research Databases Search Screen - Advanced Search Database - CINAHL
S6	(MH "Pharmacy, Retail")	Search modes - Boolean/Phrase	Interface - EBSCOhost Research Databases Search Screen - Advanced Search Database - CINAHL
S5	TX pharmacies	Search modes - Boolean/Phrase	Interface - EBSCOhost Research Databases Search Screen - Advanced Search Database - CINAHL
S4	TX pharmacy	Search modes - Boolean/Phrase	Interface - EBSCOhost Research Databases Search Screen - Advanced Search Database - CINAHL
S3	(MH "Pharmacy and Pharmacology+")	Search modes - Boolean/Phrase	Interface - EBSCOhost Research Databases Search Screen - Advanced Search Database - CINAHL
S2	TX pharmacist*	Search modes -	Interface -

		Boolean/Phrase	EBSCOhost Research Databases Search Screen - Advanced Search Database - CINAHL
S1	(MH "Pharmacists")	Search modes - Boolean/Phrase	Interface - EBSCOhost Research Databases Search Screen - Advanced Search Database - CINAHL
